# 2-Ethyl-1*H*-imidazole-4-carboxyl­ate monohydrate

**DOI:** 10.1107/S1600536811010774

**Published:** 2011-03-31

**Authors:** Shi-Jie Li, Juan-Hua Liu, Wen-Dong Song, Xiao-Fei Li, Dong-Liang Miao

**Affiliations:** a College of Food Science and Technology, Guangdong Ocean University, Zhanjiang 524088, People’s Republic of China; b College of Science, Guangdong Ocean University, Zhanjiang 524088, People’s Republic of China; c College of Agriculture, Guangdong Ocean University, Zhanjiang 524088, People’s Republic of China

## Abstract

In the title compound, C_7_H_8_N_2_O_4_·H_2_O, the imidazole N atom is protonated and one of the carboxyl­ate groups is deprontonated, forming a zwitterion. The two carboxyl groups are are approximately coplanar with the imidazole ring [O—C—C—C torsion angles = −176.8 (2) and 2.9 (4)° for one group and −4.6 (3) and 176.4 (2)° for the other] and have an intra­molecular O—H⋯O hydrogen bond between them. The water mol­ecule is linked to the organic mol­ecules *via* an N—H⋯O hydrogen bonds. Inter­molecular O—H⋯O and N—H⋯O hydrogen bonds are found in the crystal structure.

## Related literature

For our past work based on the 2-propyl-1H-imidazole-4,5-carboxyl­ate (H_3_pimda) ligand, see: Yan *et al.* (2010 ▶); Li *et al.* (2010 ▶); Song *et al.* (2010 ▶); He *et al.* (2010 ▶); Fan *et al.* (2010 ▶). For related coordination polymers based on H_3_EIDC (2-ethyl-1H-imidazole-4,5-dicarboxyl­ate), see: Wang *et al.* (2008 ▶); Zhang *et al.* (2010 ▶); Li *et al.* (2011 ▶). For the synthesis of H_3_EIDC, see: Sun *et al.* (2006 ▶).
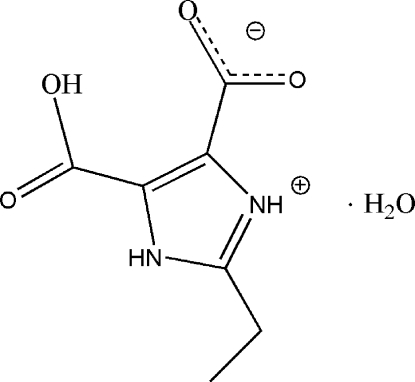


## Experimental

### 

#### Crystal data

C_7_H_10_N_2_O_5_
*M* *_r_* = 202.17
Monoclinic, 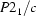

*a* = 7.6132 (6) Å
*b* = 14.3779 (16) Å
*c* = 7.9396 (8) Å
β = 97.799 (1)°
*V* = 861.04 (15) Å^3^
*Z* = 4
Mo *K*α radiation
μ = 0.13 mm^−1^
*T* = 298 K
0.50 × 0.41 × 0.40 mm

#### Data collection

Bruker SMART 1000 CCD area-detector diffractometer
Absorption correction: multi-scan (*SADABS*; Bruker, 2007 ▶) *T*_min_ = 0.936, *T*_max_ = 0.948
4224 measured reflections
1510 independent reflections
1166 reflections with *I* > 2σ(*I*)
*R*_int_ = 0.031

#### Refinement

*R*[*F* ^2^ > 2σ(*F* ^2^)] = 0.038
*wR*(*F* ^2^) = 0.106
*S* = 1.05
1510 reflections
129 parameters
3 restraints
H-atom parameters constrained
Δρ_max_ = 0.28 e Å^−3^
Δρ_min_ = −0.21 e Å^−3^

### 

Data collection: *SMART* (Bruker, 2007 ▶); cell refinement: *SAINT* (Bruker, 2007 ▶); data reduction: *SAINT*; program(s) used to solve structure: *SHELXS97* (Sheldrick, 2008 ▶); program(s) used to refine structure: *SHELXL97* (Sheldrick, 2008 ▶); molecular graphics: *SHELXTL* (Sheldrick, 2008 ▶); software used to prepare material for publication: *SHELXTL*.

## Supplementary Material

Crystal structure: contains datablocks I, 1R. DOI: 10.1107/S1600536811010774/jh2272sup1.cif

Structure factors: contains datablocks I. DOI: 10.1107/S1600536811010774/jh2272Isup2.hkl

Additional supplementary materials:  crystallographic information; 3D view; checkCIF report

## Figures and Tables

**Table 1:** Hydrogen-bond geometry (Å, °)

*D*—H⋯*A*	*D*—H	H⋯*A*	*D*⋯*A*	*D*—H⋯*A*
N1—H1⋯O4^i^	0.86	1.91	2.754 (2)	168
N2—H2⋯O1*W*^ii^	0.86	1.89	2.751 (2)	177
O2—H2*A*⋯O3	0.82	1.63	2.452 (2)	176
O1*W*—H1*W*⋯O2^iii^	0.85	2.05	2.8863 (19)	169
O1*W*—H2*W*⋯O1	0.85	2.03	2.849 (2)	163

Symmetry codes: (i) 
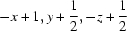
; (ii) 
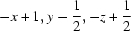
; (iii) 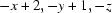.

**Fig. 1.**
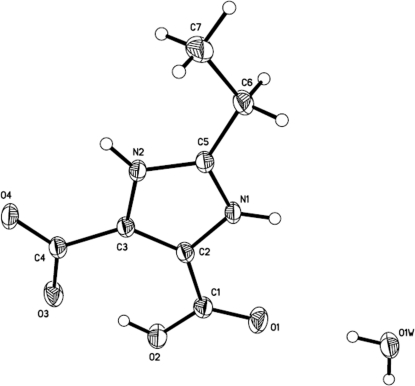
The structure of the title compound, showing the atomic numbering scheme. Non-H atoms are shown with 30% probability displacement ellipsoids.

**Fig. 2.**
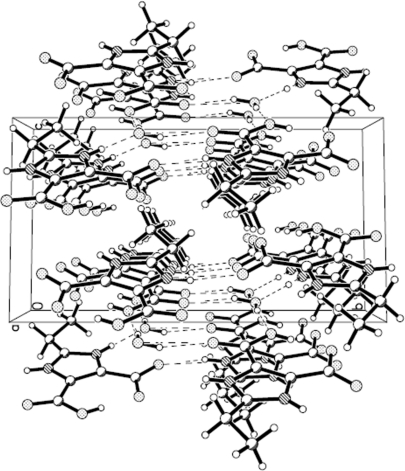
A view of the three-dimensional network constructed by O—H···O and N—H···O hydrogen bonding interactions

## supplementary crystallographic information

### Comment

4,5-imidazoledicarboxylic acid (H_3_IDC) ligand posesses great potential for coordination interactions and hydrogen bonding, can be deprotonated to generate H_2_IDC^-^, HIDC_2_^-^ and IDC_3_^-^ anions at different pH values. Up to date, it has been widely studied. 2-propyl-1H-imidazole-4,5-carboxylate (H_3_pimda) ligand as one derivative of H_3_IDC with efficient N,O-donors has been used to obtain new metal-organic complexes by our research group(Song *et al.*, 2010; Yan *et al.*, 2010; He *et al.* 2010; Fan *et al.* 2010; Li *et al.* 2010). Recently, an analogue of H_3_IDC, 2-ethyl-1H-imidazole-4,5-dicarboxylate (H_3_EIDC)ligand has also been used to construct intriguing coordination polymers (Wang *et al.*, 2008; Zhang *et al.* , 2010; Li *et al.*, 2011;). However, the crystal structure of H_3_EIDC ligand has not been determined. Considering that in mind, we focus on obtaining the crystal and its crystal structure will be reported here.

As illustrated in Fig. 1, the title compound, (C_7_H_8_N_2_O_4_).H_2_O, crystallizes as a zwitterion in which the imidazole N atom is protonated, one of the carboxylate groups is deprontonated. The two carboxyl groups are approximately coplanar with the imidazole ring, as indicated by the fact that the O1—C1—C2—C3 and O2—C1—C2—C3 torsion angles are -176.8 (2) ° and 2.9 (4) °, respectively; the O3—C4—C3—C2 and O4—C4—C3—C2 torsion angles are -4.6 (3) ° and 176.4 (2) °, respectively. The solvent water molecules are linked to the organic ligands via N—H···O and O—H···O hydrogen bonds(Table 1), which stabilize the three-dimensional network(Fig. 2).

### Experimental

The organic molecule powder was abtained from 2-ethylbenzimidazole according to a literature procedure (Sun *et al.* 2006), then the 2-ethyl-1H-imidazole-4,5-dicarboxylate(0.5 mmol, 0.9 g) was disolved in 15 ml of H_2_O solution with the pH of 6 adjusted by NaOH, colorless crystals was obtained by slow evaporation of the solvent at room temperature.

### Refinement

H atoms of the water molecule were located in a difference Fourier map and refined as riding with an O—H distance restraint of 0.84 (1) Å, with *U*_iso_(H) = 1.5 *U*_eq_. The H···H distances within the water molecules were restraint to 1.39 (1) Å. Carboxyl H atoms were located in a difference map but were refined as riding on the parent O atoms with with O—H = 0.82 Å with *U*_iso_(H) = 1.5 *U*_eq_(O). Carbon and nitrogen bound H atoms were placed at calculated positions and were treated as riding on the parent C or N atoms with C—H = 0.96 (methyl), 0.97 (methylene) and N—H = 0.86 Å, *U*_iso_(H) = 1.2 or 1.5 *U*_eq_(C, N).

### Crystal data

**Table:**

C_7_H_10_N_2_O_5_	*F*(000) = 424
*M**_r_* = 202.17	*D*_x_ = 1.560 Mg m^−^^3^
Monoclinic, *P*2_1_/*c*	Mo *K*α radiation, λ = 0.71073 Å
Hall symbol: -P 2ybc	Cell parameters from 1702 reflections
*a* = 7.6132 (6) Å	θ = 2.5–25.9°
*b* = 14.3779 (16) Å	µ = 0.13 mm^−^^1^
*c* = 7.9396 (8) Å	*T* = 298 K
β = 97.799 (1)°	Block, colorless
*V* = 861.04 (15) Å^3^	0.50 × 0.41 × 0.40 mm
*Z* = 4	

### Data collection

**Table:**

Bruker SMART 1000 CCD area-detector diffractometer	1510 independent reflections
Radiation source: fine-focus sealed tube	1166 reflections with *I* > 2σ(*I*)
graphite	*R*_int_ = 0.031
φ and ω scans	θ_max_ = 25.0°, θ_min_ = 2.7°
Absorption correction: multi-scan (*SADABS*; Bruker, 2007)	*h* = −9→8
*T*_min_ = 0.936, *T*_max_ = 0.948	*k* = −17→13
4224 measured reflections	*l* = −9→7

### Refinement

**Table:**

Refinement on *F*^2^	Secondary atom site location: difference Fourier map
Least-squares matrix: full	Hydrogen site location: inferred from neighbouring sites
*R*[*F*^2^ > 2σ(*F*^2^)] = 0.038	H-atom parameters constrained
*wR*(*F*^2^) = 0.106	*w* = 1/[σ^2^(*F*_o_^2^) + (0.047*P*)^2^ + 0.3916*P*] where *P* = (*F*_o_^2^ + 2*F*_c_^2^)/3
*S* = 1.05	(Δ/σ)_max_ < 0.001
1510 reflections	Δρ_max_ = 0.28 e Å^−^^3^
129 parameters	Δρ_min_ = −0.21 e Å^−^^3^
3 restraints	Extinction correction: *SHELXL*, Fc^*^=kFc[1+0.001xFc^2^λ^3^/sin(2θ)]^-1/4^
Primary atom site location: structure-invariant direct methods	Extinction coefficient: 0.060 (5)

### Special details

**Table:**

Geometry. All esds (except the esd in the dihedral angle between two l.s. planes) are estimated using the full covariance matrix. The cell esds are taken into account individually in the estimation of esds in distances, angles and torsion angles; correlations between esds in cell parameters are only used when they are defined by crystal symmetry. An approximate (isotropic) treatment of cell esds is used for estimating esds involving l.s. planes.
Refinement. Refinement of F^2^ against ALL reflections. The weighted R-factor wR and goodness of fit S are based on F^2^, conventional R-factors R are based on F, with F set to zero for negative F^2^. The threshold expression of F^2^ > 2sigma(F^2^) is used only for calculating R-factors(gt) etc. and is not relevant to the choice of reflections for refinement. R-factors based on F^2^ are statistically about twice as large as those based on F, and R- factors based on ALL data will be even larger.

### Fractional atomic coordinates and isotropic or equivalent isotropic displacement parameters (Å^2^)

**Table:**

	*x*	*y*	*z*	*U*_iso_*/*U*_eq_	
N1	0.4705 (2)	0.42162 (11)	0.2301 (2)	0.0314 (4)	
H1	0.4860	0.4805	0.2208	0.038*	
N2	0.3607 (2)	0.29056 (11)	0.2927 (2)	0.0305 (4)	
H2	0.2932	0.2499	0.3308	0.037*	
O1	0.7740 (2)	0.46097 (10)	0.0811 (2)	0.0517 (5)	
O2	0.8238 (2)	0.31103 (10)	0.0434 (2)	0.0457 (5)	
H2A	0.7770	0.2619	0.0653	0.069*	
O3	0.6974 (2)	0.16119 (9)	0.1124 (2)	0.0433 (4)	
O4	0.4800 (2)	0.11124 (9)	0.2502 (2)	0.0436 (4)	
O1W	0.8455 (2)	0.65550 (10)	0.0862 (2)	0.0501 (5)	
H1W	0.9476	0.6582	0.0543	0.075*	
H2W	0.8189	0.5995	0.1057	0.075*	
C1	0.7364 (3)	0.37964 (14)	0.0950 (3)	0.0356 (5)	
C2	0.5789 (3)	0.35434 (13)	0.1777 (2)	0.0295 (5)	
C3	0.5090 (3)	0.27125 (13)	0.2173 (2)	0.0282 (5)	
C4	0.5648 (3)	0.17372 (13)	0.1935 (3)	0.0314 (5)	
C5	0.3383 (3)	0.38238 (13)	0.2972 (3)	0.0310 (5)	
C6	0.1908 (3)	0.43371 (15)	0.3579 (3)	0.0466 (6)	
H6A	0.1212	0.4632	0.2610	0.056*	
H6B	0.2403	0.4827	0.4340	0.056*	
C7	0.0694 (3)	0.37559 (17)	0.4485 (3)	0.0528 (7)	
H7A	0.0221	0.3257	0.3758	0.079*	
H7B	−0.0259	0.4135	0.4773	0.079*	
H7C	0.1345	0.3504	0.5503	0.079*	

### Atomic displacement parameters (Å^2^)

**Table:**

	*U*^11^	*U*^22^	*U*^33^	*U*^12^	*U*^13^	*U*^23^
N1	0.0332 (9)	0.0177 (8)	0.0455 (11)	0.0005 (7)	0.0140 (8)	0.0015 (7)
N2	0.0316 (9)	0.0200 (8)	0.0425 (10)	−0.0005 (6)	0.0145 (8)	0.0011 (7)
O1	0.0503 (10)	0.0288 (9)	0.0821 (13)	−0.0062 (7)	0.0310 (9)	0.0027 (8)
O2	0.0437 (9)	0.0295 (8)	0.0707 (11)	0.0011 (7)	0.0321 (8)	0.0009 (7)
O3	0.0479 (9)	0.0275 (8)	0.0601 (10)	0.0068 (6)	0.0272 (8)	−0.0009 (7)
O4	0.0460 (9)	0.0202 (7)	0.0690 (11)	0.0006 (6)	0.0236 (8)	0.0029 (7)
O1W	0.0479 (10)	0.0283 (8)	0.0813 (12)	−0.0006 (7)	0.0344 (9)	−0.0023 (8)
C1	0.0344 (11)	0.0281 (11)	0.0466 (13)	0.0005 (9)	0.0137 (10)	0.0017 (9)
C2	0.0306 (11)	0.0234 (10)	0.0355 (11)	0.0029 (8)	0.0087 (9)	−0.0001 (8)
C3	0.0296 (10)	0.0222 (10)	0.0340 (11)	0.0015 (8)	0.0090 (9)	−0.0005 (8)
C4	0.0337 (11)	0.0227 (10)	0.0389 (12)	0.0023 (8)	0.0092 (9)	−0.0002 (9)
C5	0.0313 (11)	0.0222 (10)	0.0414 (12)	0.0011 (8)	0.0124 (9)	0.0005 (9)
C6	0.0433 (13)	0.0273 (12)	0.0751 (17)	0.0048 (9)	0.0295 (13)	−0.0017 (11)
C7	0.0468 (14)	0.0451 (14)	0.0734 (17)	0.0080 (11)	0.0334 (13)	0.0037 (13)

### Geometric parameters (Å, °)

**Table:**

N1—C5	1.327 (2)	O1W—H2W	0.8501
N1—C2	1.372 (2)	C1—C2	1.488 (3)
N1—H1	0.8600	C2—C3	1.362 (3)
N2—C5	1.332 (3)	C3—C4	1.485 (3)
N2—C3	1.376 (2)	C5—C6	1.478 (3)
N2—H2	0.8600	C6—C7	1.500 (3)
O1—C1	1.212 (2)	C6—H6A	0.9700
O2—C1	1.288 (2)	C6—H6B	0.9700
O2—H2A	0.8200	C7—H7A	0.9600
O3—C4	1.281 (2)	C7—H7B	0.9600
O4—C4	1.227 (2)	C7—H7C	0.9600
O1W—H1W	0.8500		
			
C5—N1—C2	110.02 (16)	O4—C4—C3	118.07 (17)
C5—N1—H1	125.0	O3—C4—C3	117.11 (17)
C2—N1—H1	125.0	N1—C5—N2	107.67 (16)
C5—N2—C3	109.11 (15)	N1—C5—C6	124.74 (17)
C5—N2—H2	125.4	N2—C5—C6	127.55 (17)
C3—N2—H2	125.4	C5—C6—C7	115.01 (18)
C1—O2—H2A	109.5	C5—C6—H6A	108.5
H1W—O1W—H2W	110.3	C7—C6—H6A	108.5
O1—C1—O2	124.86 (19)	C5—C6—H6B	108.5
O1—C1—C2	119.35 (18)	C7—C6—H6B	108.5
O2—C1—C2	115.80 (17)	H6A—C6—H6B	107.5
C3—C2—N1	106.15 (16)	C6—C7—H7A	109.5
C3—C2—C1	132.84 (18)	C6—C7—H7B	109.5
N1—C2—C1	121.01 (17)	H7A—C7—H7B	109.5
C2—C3—N2	107.05 (16)	C6—C7—H7C	109.5
C2—C3—C4	132.14 (17)	H7A—C7—H7C	109.5
N2—C3—C4	120.81 (17)	H7B—C7—H7C	109.5
O4—C4—O3	124.82 (17)		
			
C5—N1—C2—C3	−0.7 (2)	C5—N2—C3—C4	−179.30 (18)
C5—N1—C2—C1	179.02 (18)	C2—C3—C4—O4	176.4 (2)
O1—C1—C2—C3	−176.8 (2)	N2—C3—C4—O4	−3.4 (3)
O2—C1—C2—C3	2.9 (4)	C2—C3—C4—O3	−4.6 (3)
O1—C1—C2—N1	3.5 (3)	N2—C3—C4—O3	175.56 (18)
O2—C1—C2—N1	−176.74 (19)	C2—N1—C5—N2	1.3 (2)
N1—C2—C3—N2	−0.1 (2)	C2—N1—C5—C6	−176.3 (2)
C1—C2—C3—N2	−179.8 (2)	C3—N2—C5—N1	−1.3 (2)
N1—C2—C3—C4	−179.9 (2)	C3—N2—C5—C6	176.2 (2)
C1—C2—C3—C4	0.4 (4)	N1—C5—C6—C7	−172.7 (2)
C5—N2—C3—C2	0.9 (2)	N2—C5—C6—C7	10.2 (4)

### Hydrogen-bond geometry (Å, °)

**Table:**

*D*—H···*A*	*D*—H	H···*A*	*D*···*A*	*D*—H···*A*
N1—H1···O4^i^	0.86	1.91	2.754 (2)	168
N2—H2···O1W^ii^	0.86	1.89	2.751 (2)	177
O2—H2A···O3	0.82	1.63	2.452 (2)	176
O1W—H1W···O2^iii^	0.85	2.05	2.8863 (19)	169
O1W—H2W···O1	0.85	2.03	2.849 (2)	163

Symmetry codes: (i) −*x*+1, *y*+1/2, −*z*+1/2; (ii) −*x*+1, *y*−1/2, −*z*+1/2; (iii) −*x*+2, −*y*+1, −*z*.
